# Selecting the optimal MIC for five patient-reported outcome measures in patients with upper extremity injuries, using a systematic search and step-by-step decision tree

**DOI:** 10.1007/s11136-025-04152-1

**Published:** 2026-03-13

**Authors:** S. G. J. Suus van Bruggen, F. L. Floortje Opperman, E. P. Ilse Jansma, C. M. Charlotte Lameijer, F. W. Frank Bloemers, C. B. Caroline Terwee

**Affiliations:** 1 Department of Trauma Surgery, Amsterdam UMC, Amsterdam, The Netherlands; 2 Amsterdam Movement Sciences, Musculoskeletal Health, Amsterdam, The Netherlands; 3 Amsterdam UMC Location VUmc, Boelelaan, Amsterdam, The Netherlands; 4 Epidemiology and Data Science, Amsterdam UMC Location Vrije Universiteit, Amsterdam, The Netherlands; 5 Amsterdam Public Health Research Institute, Methodology, Amsterdam, the Netherlands

**Keywords:** DASH, MHQ, PRWE, PROMIS upper extremity, Minimal important change, Upper extremity injury

## Abstract

**Purpose:**

To illustrate how optimal MIC values can be selected for PROMs using a systematic step-by-step approach.

**Methods:**

A systematic search was performed in PubMed, Embase and Cochrane, until June 2024 to identify all studies that estimated the MIC for the following PROMs in patients with upper extremity injuries: DASH, Q-DASH, MHQ, PRWE and PROMIS-UE. Credibility of the MIC values was examined using the MIC credibility instrument. The optimal MIC value was selected using a published systematic step-by-step approach, taking the credibility of the MIC studies, the correlation of the PROM change score with the anchor, the consistency of MIC values, and contextual factors (e.g. type of intervention) into account.

**Results:**

Eleven studies were included. Eight studies scored high on the core credibility items, but all studies scored low on the additional criteria, regarding the appropriateness of the time interval and the correlation between the anchor and (change in) PROM scores. The most credible estimates per PROM were not consistent and there were not enough estimates per PROM to study contextual factors. The following optimal MIC values (among ranges of MIC values found) were selected: DASH 9.4 (6.7–13.0), Q-DASH 7.4 (3.5–11.7), PRWE 11.5 (9.2–56.0), and PROMIS-UE v1.2 4.7 (4.6–4.8). No studies were found on the MIC of the MHQ in Upper Extremity Injury (UEI) patients.

**Conclusion:**

This study serves as an example of how optimal MIC values can be carefully selected for a given PROM if multiple MIC values are reported in the literature.

**Supplementary Information:**

The online version contains supplementary material available at 10.1007/s11136-025-04152-1.

## Introduction

To facilitate the use of patient-reported outcome measures (PROMs) in research and clinical practice, PROM scores and change in scores should be easy to interpret. One of the most often assessed aspects of interpretability is the Minimal Important Change (MIC). The MIC has been defined as ‘the smallest change in score in the construct to be measured which patients perceive as important’ [1]. The MIC can be used in clinical practice to facilitate shared-decision making by informing patients about the expected effects of treatments, in relation to what patients, on average, consider an important change, and in research to determine the number of patients who achieved a MIC after treatment (responder analysis) [1, 2].

In the literature, a wide range of MIC values are often reported and recommended for the same PROM. These variations may be attributed to differences in study methodology and quality. For example, the MIC can be estimated with different methods, often distinguished into distribution-based methods and anchor-based methods [3]. Distribution-based methods rely on statistical parameters, and reflect the measurement error or standard deviation of a metric. These methods do not represent what changes in scores patients consider important [1, 4, 5]. Anchor-based methods relate PROM change scores to an external criterion; the anchor (also called transition rating anchor or transition item) [5, 6]. Anchors are often single questions used to define what change is important for the individual patient. Several anchor-based methods for MIC estimation are being used; the mean (difference or) change method and de ROC (Receiver Operating Characteristic) method are most often used, but have important methodological shortcomings. The predictive modelling method, and longitudinal item response theory (IRT) or confirmatory factor analysis (CFA) methods are most promising, but not yet often used [1, 6–9].

The variation in published MIC values of PROMs creates uncertainty regarding which MIC values should be considered most appropriate. Recently, Wang et al. proposed a systematic, step-by-step selection approach for selecting an optimal MIC value per PROM. This approach aims to explain the variability in MIC values for the PROM of interest by the methodological study quality and contextual factors, and where appropriate, takes the median of selected estimates in a relatively narrow range as the optimal MIC value [10].

While the method proposed by Wang et al. appears to be a valuable approach to obtain more valid MIC values, it has not yet been applied to select optimal MIC values of PROMs, aside from several illustrative examples provided by Wang et al. About 25% of all visits to the Emergency Department are due to Upper Extremity Injuries (UEIs) [11]. UEIs have a major impact on physical health and quality of life, especially the ability to perform daily activities and work, and participate in social activities, as well as on health care costs [12, 13]. The objective of this paper is therefore to illustrate how to select optimal MIC values of PROMs, using five frequently used PROMs for measuring upper extremity function: the Disability of the Arm, Shoulder and Hand (DASH) questionnaire [14], the Quick-Disability of the Arm, Shoulder and Hand (Q-DASH) questionnaire [14], the Patient-Rated Wrist Evaluation (PRWE) questionnaire [15], the Michigan Hand Outcomes Questionnaire (MHQ), and the Patient-Reported Outcomes Measurement Information System (PROMIS) Upper Extremity item bank (PROMIS-UE v1.2, later updated to v2.0 and v2.1) [16]. In addition, this paper illustrates how important valid, reliable and interpretable PROMs are to measure the impact of UEIs and the effects of treatment.

## Methods

### Literature search

A systematic literature search was performed following the PRISMA guidelines, in PubMed, Embase and the Cochrane Library, until June 2024, to identify all published studies on the MIC of the five PROMs. The search strategy was developed in collaboration with an information specialist (IJ), specialized in PROM searches. The databases were searched with combinations of MeSH, title and abstract terms regarding the selected PROMs (DASH, Q-DASH, PRWE, MHQ and PROMIS-UE) and search terms for the MIC (Online Appendix 1). No MeSH terms are available for the MIC. An updated selection of relevant search terms from the validated search filter for finding studies on measurement properties was used [17]. Because there is lack of consensus in the literature about the terminology for MIC, other search terms were used as well; Minimal Important Change, Minimal Clinically Important Difference, and Minimal Important Difference. In addition, because terminology and methods to estimate the MIC and the minimal detectable change (i.e. measurement error) are often confused in literature, search terms for minimal detectable change were also included (Online Appendix 2). References were checked for additional articles. A protocol was not prepared.

### Inclusion and exclusion criteria

Eligible were studies including adult patients suffering uni- or bilateral UEIs. Study populations were included and categorized as UEI if baseline PROMs were completed less than 1 year following injury. Study populations who completed baseline PROMs ≥ 1 year following injury, patients with typical chronic upper extremity disorders (UED) or combined UEI and/or UED conditions were excluded. An inclusion cut-off of within 1-year post-injury was applied to classify upper extremity conditions as either acute or chronic. This criterion ensured a clear distinction between acute and chronic injuries (UEI vs UED) and enabled us to specifically target acute UEI, which represents the predominant caseload in trauma surgery practice. Studies were included if they estimated the MIC of at least one of the selected PROMs of interest: DASH, Q-DASH, PRWE, MHQ or PROMIS-UE item bank, short form or CAT, in any version or language, in patients with UEI. A description of the PROM characteristics is presented in Online Appendix 3. Studies written in languages other than English or Dutch and abstracts were excluded.

### Study selection

Two reviewers (SB and FO) independently screened titles and abstracts. Full texts of the selected abstracts were reviewed independently by the same reviewers. Differences were discussed and in case of persistent disagreement, a third reviewer (CL) was consulted to reach consensus. All MIC values reported per PROM were extracted from the included papers. In addition, information was extracted on study population (localization, injury, age), sample size, follow-up time, and MIC methods used. Data was extracted by one author (SB).

### Credibility of MIC values

To assess the trustworthiness of the MIC values, the credibility instrument developed by Devji et al. [18], was used (Table 1). This instrument includes five core criteria regarding the quality of the methods used to estimate the MIC (including the validity of the anchor and precision of the MIC values), as well as four additional criteria regarding the correlations between the anchor and (change in) PROM scores and the appropriateness of the time elapsed between baseline and follow-up measurements. Devji et al. argued that patients have considerable difficulty recalling previous health states. Studies showed that patients can often remember previous states for up to four weeks. Therefore, they consider MIC estimates based on studies with a longer time interval less credible [18]. The MIC credibility instrument does not have a specific scoring system to distinguish between high or low quality, but acts as a tool to provide supporting information for someone’s judgment on each credibility item. In this review, credibility was defined by the reviewers as predominantly high credibility when at least three of the five core items were rated as high credibility (‘yes’, ‘definitely yes’ or ‘to a great extent’). Only the most credible studies, meaning the studies in which at least three of the five core items were rated as high credibility, were used to select the optimal MIC values. Two reviewers (SB and FO) independently scored the quality of each included study. Difference in scoring between the two reviewers was resolved with discussion and in consultation with a third reviewer (CBT) to reach consensus.

**Table 1:** Credibility instrument for judging the trustworthiness of the MIC [1]

Signalling question	Response options	
	High credibility	Low credibility
*Core criteria*		
1. Is the patient or necessary proxy responding directly to both the PROM and the anchor?	Yes	No/impossible to tell
2. Is the anchor easily understandable and relevant for patients or necessary proxy?	Definitely yes/to a great extent	Definitely no/not so much/impossible to tell
3. Has the anchor shown good correlation with the PROM?		
4. Is the MIC precise?		
5. Does the threshold or difference between groups on the anchor used to estimate the MIC reflect a small but important difference?		
*Additional criteria for transition rating anchors*		
6. Is the amount of elapsed time between baseline and follow-up measurement for MIC estimation optimal?	Definitely yes/to a great extent	Definitely no/not so much/impossible to tell
7. Does the transition item have a satisfactory correlation with the PROM score at follow-up?		
8. Does the transition item correlate with the PROM score at baseline?		
9. Is the correlation of the transition item with the PROM change score appreciably greater than the correlation of the transition item with the PROM score at follow-up?		

Devji et al. [18]

### Data synthesis

The systematic step-by-step approach, developed by Wang et al., was used to select an optimal MIC for the PROM [10]. The optimal MIC value was selected using a flow chart proposed by Wang et al., taking the credibility of the MIC studies, the correlation of the PROM change score with the anchor, the consistency of MIC values, and contextual factors (e.g. type of intervention) into account [10].

The study was reported according to the PRISMA guideline [19]. A protocol was not prepared.

## Results

### Study selection

**Fig. 1:**
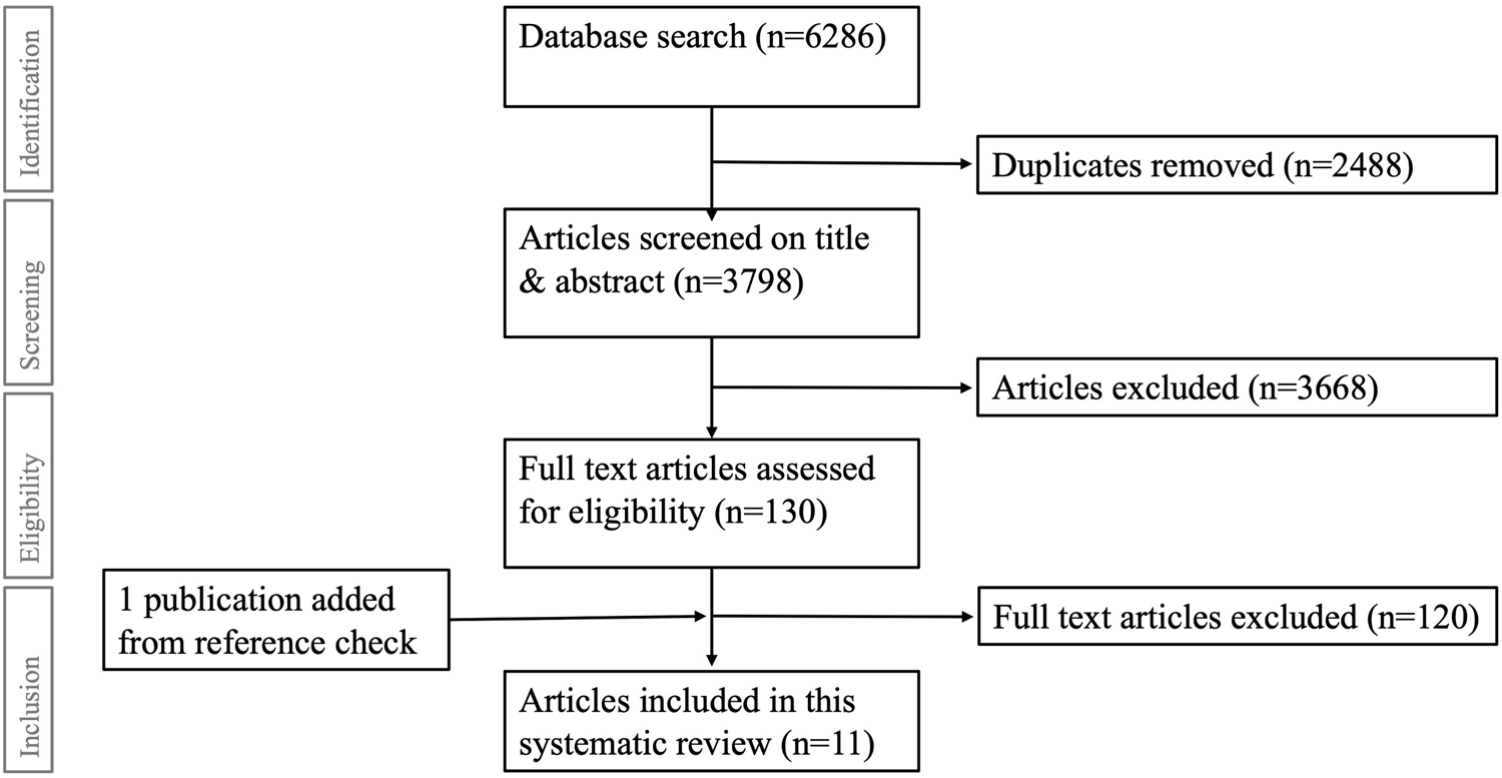
Flow-chart of the study selection

Out of a total of 6286 articles, 11 studies were included (Fig. 1). Three studies reported MIC values for the DASH, 3 for the Q-DASH, 5 for the PRWE, none for the MHQ, and 1 for the PROMIS-UE. A total of 1591 patients (64% female) were included (20–390 patients per study) (Table 2A–D). Age varied widely (11–89) with a mean of 54.2 years.

**Table 2:** Literature search results per PROM: DASH (2A), Q-DASH (2B), PRWE (2C), PROMIS-UE (2D)

Author, year	Credibility scores	Localization	Injury	N	Mean age (range)	Mean change score (SD)	Follow up	MIC method	MIC
*DASH (2A)*									
Ibounig et al. 2022 [20]	C 4/5 A 0/4 T 4/9	Shoulder	Humeral shaft fracture ORIF vs bracing	124 (surgery 47, bracing 77) ♂ 70,♀ 54	47 (SD 17)	–	6w, 3m, 6m, 12m, 24m	ROC MCM MDCM PM	− 6.7 − 11.2 − 6.8 − 9.4
Mahabier et al. 2017 [21]	C 3/5 A 0/4 T 3/9	Shoulder	Humeral shaft fracture	140 ♂ 63,♀ 77	58 (–)	27.8 (17.1)	6w, 12m	ROC SEM	6.7 6.9
van de Water et al. 2014 [22]	C 3/5 A 1/4 T 4/9	Shoulder	Proximal humeral fracture	20 ♂4,♀ 16	68.1 (50–86)	17.4	6w, 12w	MCM 0.5 SD	13.0 8.1
*Q-DASH (2B)*									
Iordens et al. 2017 [23]	C 3/5 A 0/4 T 3/9	Elbow	Elbow dislocation	100 ♂ 42,♀ 58	46 (median) (IQR 32–59)	11.1 (12.8)	B, 6w-12m	ROC SD^1^ SEM	3.5 6.2 4.4
Randall et al. 2021 [24]	C 3/5 A 0/4 T 4/9	Elbow	e.g. non-operative fractures, ORIF for distal humerus/radial head/olecranon fracture, distal biceps repair	110 ♂ 58,♀ 52 (MDCM 57pt, 0.5SD 114pt)	46.4 (11–86)	11.9	B, 2-6w	MDCM 0.5 SD	5.3 11.7
Wanstrom et al. 2024 [25]	C 3/5 A 1/4 T 6/9	Elbow	e.g. proximal radius/ olecranon fracture, terrible triad, elbow dislocation	97 ♂ 67,♀ 33	52 (SD 18.2)	7.2	3-5m, 3.5m+3w	MCM	7.4
*PRWE (2C)*									
Huyke-Hernández et al. 2023 [26]	C 4/5 A 0/4 T 4/9	Hand/wrist	DRF—> volar plate fixation	131 ♂ 20,♀ 111	59 (SD 13.4)	B -> 6w: -37.5 (± 23.4) B -> 12w: -50.6 (± 22.3)	B, 6w, 12w	MDCM	6w: 43.1 ± 18.0 12w: 56.0 ± 20.0
Larose et al. 2024 [27]	C 1/5 A 0/4 T 1/9	Hand/wrist	DRF—> ORIF	390 ♂ 125,♀ 265	51 (17–93)	19.0	B, 6m, 12m, 5y	0.5 SD	9.2
McCreary et al. 2020 [28]	C 1/5 A 0/4 T 1/9	Hand/wrist	DRF	197 ♂ 48,♀ 149	57 (-)	0-6w: 23.4 0-12w: 36 6-12w: 12.6	B, 6w, 12w	MCM SD^1^	Mean MIC: 26 A 0–6w: 26.8 A 0–12w: 42.6 A 6–12w: 14.6 D 0w: 10.9
Perez-Ubeda et al. 2024 [29]	C 1/5 A 0/4 T 1/9	Hand/wrist	DRF—> volar plate fixation with/without arthroscopy	180 ♂43, ♀137	59.0 (20–89)	–	4w, 12m	0.5 SD	12.8
Walenkamp et al. 2015 [30]	C 3/5 A 0/4 T 3/9	Hand/wrist	DRF	102 ♂31, ♀71	59 (–)	Tot: - Func: 10.8	B (6-12w), 12-52w	ROC	Tot: 11.5 Func: 10
*PROMIS-UE (2D)*									
Randall et al. 2021 [24] *v1.2 CAT*	C 4/5 A 0/4 T 4/9	Elbow	e.g. non-operative fractures, ORIF for distal humerus/radial head/olecranon fracture, distal biceps repair	87 ♂46, ♀41 (MDCM 43pt, 0.5SD 91pt)	45.7 (11–86)	3.1 (9.1)	B, 2-6w	MDCM 0.5 SD	4.6 4.8

C = credibility core score, A = credibility additional score, T = credibility total score

DRF = distal radius fracture. ORIF = open reduction and internal fixation

B = baseline, w = week, m = month, y = year

ROC = receiver operating characteristic method. MCM = mean change method. MDCM = mean difference of change method. PM = predictive modelling method. SD^1^ = standard deviation based methods.

### Credibility of MIC values

MIC values qualified as predominantly high credibility were found for the DASH in 3 out of 3 articles, for the Q-DASH in 3 out of 3 articles, for the PRWE in 2 out of 5 articles, and for the PROMIS-UE in 1 out of 1 article. Additional criteria regarding the correlation between PROM and anchor were rated as poor for all studies (Table 2A–D and Online Appendix 4) [18].

#### DASH

**Fig. 2:**
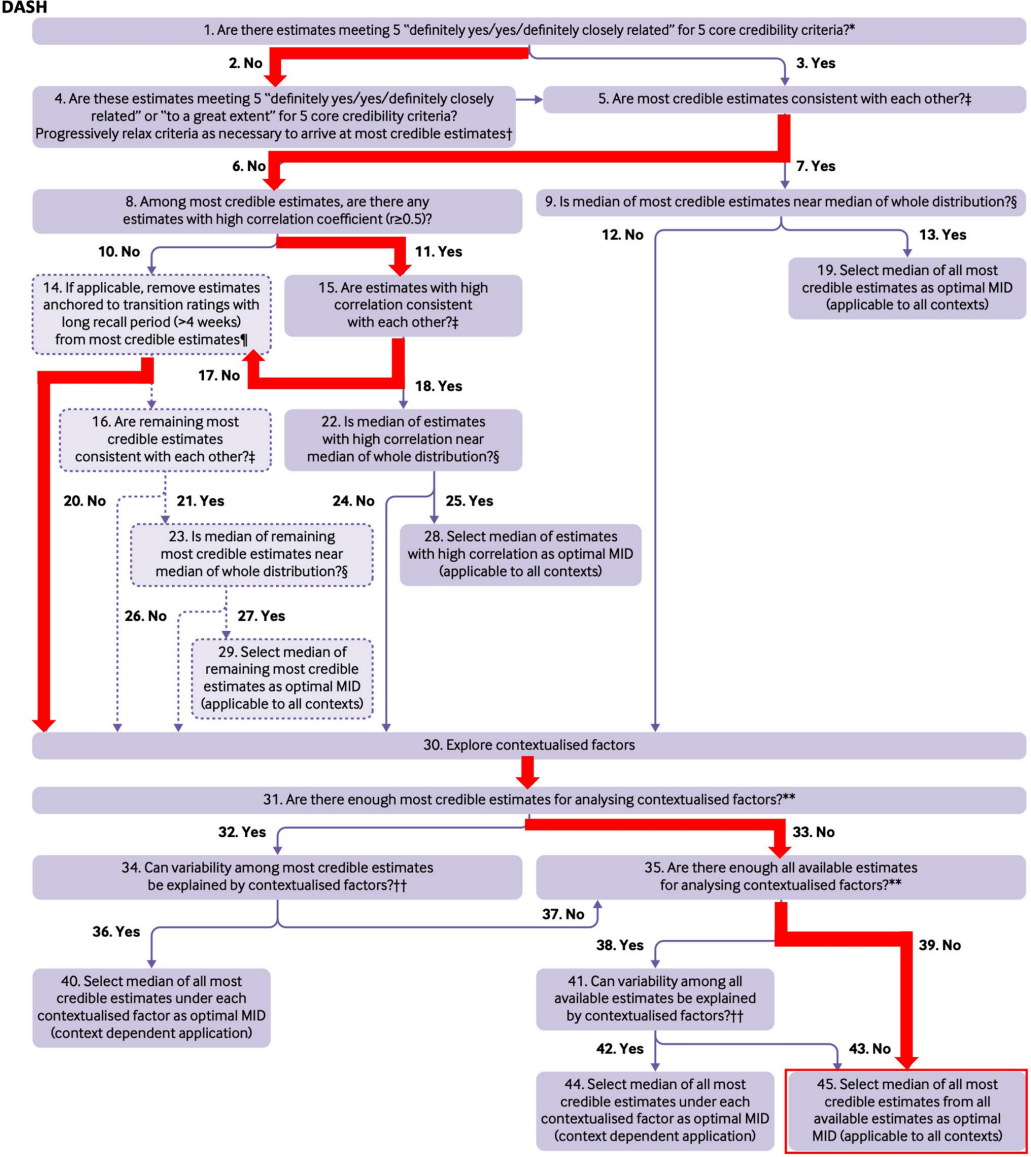
Flow-chart for selecting the optimal MIC for the DASH [10]. Q8: PROM correlation >0.5 → only Ibounig et al*.* See Table 2A and Online Appendix 4. Q30: Different methods (ROC, MCM, MDCM, PM). See Table 2A. Q45: Most appropriate method → predictive modelling method (Ibounig et al*.*) = 9.4.

Three studies reported the MIC for the DASH, with MIC values ranging from 6.7 to 13.0 (on a metric from 0 to 100, no disability to most severe disability) (Table 2A) [20–22]. The studies had credibility scores of 3 and 4 out of 5 core items. The most credible estimates were not consistent and there were not enough estimates to study contextual factors. The optimal MIC value was therefore based on the study that used the most appropriate method (predictive modeling) [20], resulting in an optimal MIC value of 9.4 on a scale from 0 to 100 (higher scores indicating more disability) (Table 3 and Fig. 2).

**Table 3:** Recommended MIC thresholds

	Recommended MIC threshold	Total MIC-ranges
DASH	9.4	6.7–13.0
Q-DASH	7.4	3.5–11.7
PRWE	11.5	9.2–56.0
PROMIS-UE v1.2	4.7	4.6–4.8

#### Q-DASH

**Fig. 3:**
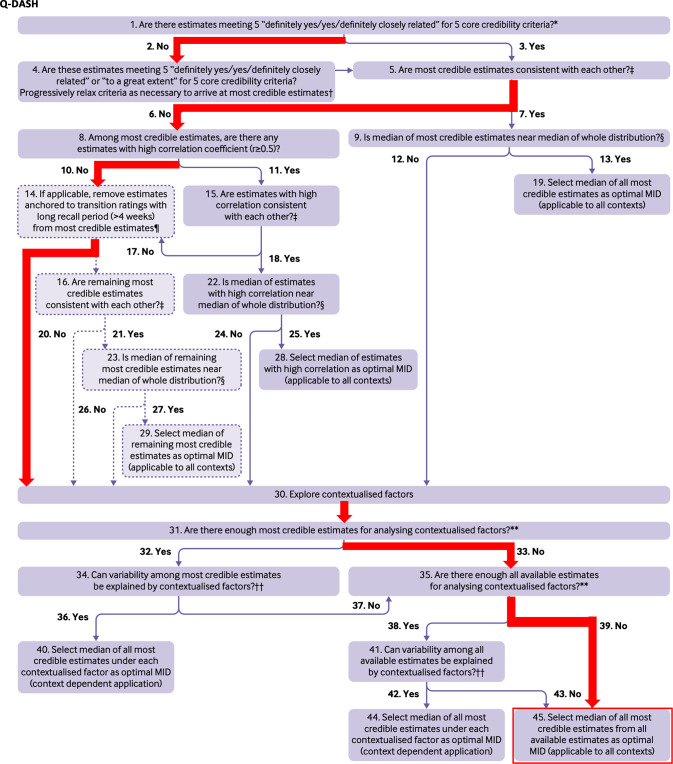
Flow-chart for selecting the optimal MIC for the Q-DASH [10]. Q14: Iordens et al. and Randall et al. both have a time interval between baseline and follow-up of  >4 weeks, see Table 2B. Both were removed; only Wanstrom et al. was kept. Q45: Wanstrom et al. reported a MIC of 7.4.

Three studies reported the MIC of the Q-DASH, with MIC values ranging from 3.5 to 11.7 (on the same metric as the DASH) (Table 2B) [23–25]. All studies had credibility scores of 3 out of 5 core items. The most credible estimates were not consistent. The MIC values of two studies with a recall period >4 weeks were ignored. The optimal MIC value was based on the study by Wänström et al. [25], resulting in an optimal MIC value of 7.4 on a scale from 0–100 (higher scores indicating more disability) (Table 3 and Fig. 3).

#### PRWE

**Fig. 4:**
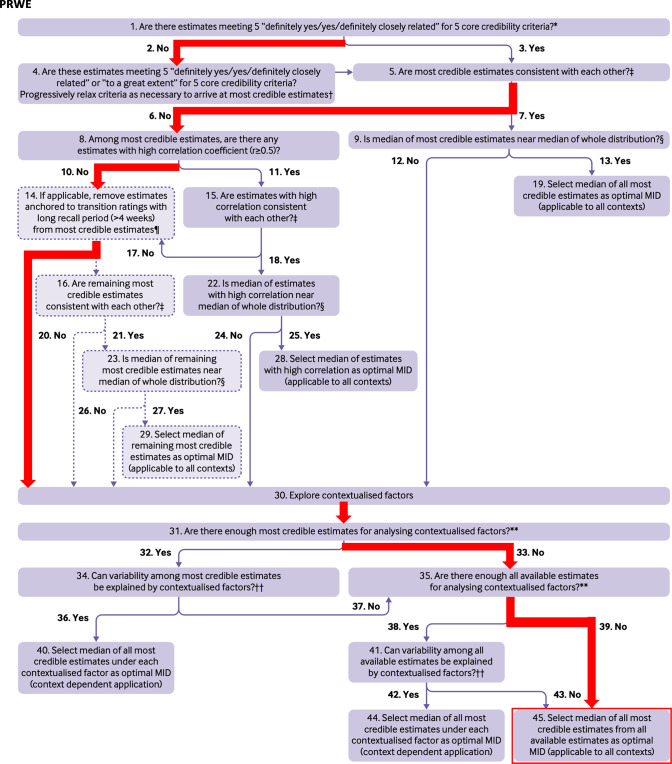
Flow-chart for selecting the optimal MIC for the PRWE [10]. Q4: Huyke et al. and Walenkamp et al. were selected. Q14: Both studies had a time interval between baseline and follow-up of  >4 weeks, so none of them was removed. Q30: Both studies focussed on patients with distal radius fractures. Huyke et al. compared baseline (day 0) with 6 and 12 weeks after injury, using the mean change method. Logically patients improved at 6 and 12 weeks after injury compared to the day of injury. Walenkamp et al. also used suboptimal follow-up moments, while the ROC anchor method is more appropriate. Q45: The MICs of Huyke et al. were excluded from selecting the median, as they were impropably high. This resulted in a MIC of 11.5 for the PRWE total score based on Walenkamp et al.

Five studies reported the MIC of the PRWE. All studies reported MIC values for the total PRWE score. One study also reported MIC values for subscales, but this study was conducted in Dutch patients and subscales are not recommended for use for the Dutch version, so these MIC values were not extracted. The MIC values for the total scores ranged from 9.2 to 56.0 (on a metric from 0 to 100, no disability and pain to most disability and unbearable pain) (Table 2C) [26–30]. Only two studies had credibility scores of 3 and 4 out of 5 core items [26, 30], the other three studies scored only 1 core item [27–29]. The estimates from these two studies were not consistent. Both studies had a recall period > 4 weeks, but since these were the only credible estimates, both studies were still considered. The MIC values reported by Huyke et al. [26] were subsequently ignored because they were improbably high. The optimal MIC value was therefore only based on the study of Walenkamp et al. [30], resulting in an optimal MIC value of 11.5 on a scale from 0 to 100 (higher scores indicating more disability) (Table 3 and Fig. 4).

#### MHQ

No studies were found on the MIC of the MHQ in UEI patients.

#### PROMIS-UE

**Fig. 5:**
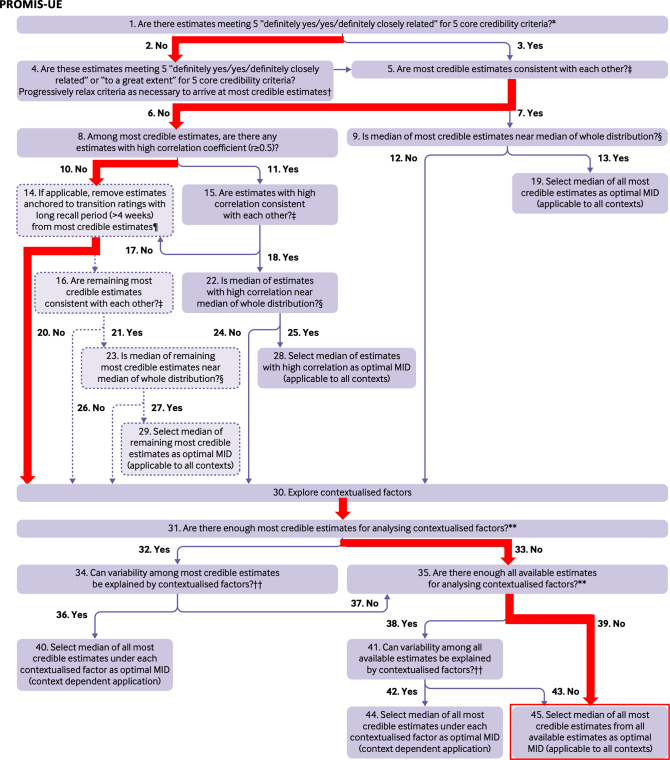
Flow-chart for selecting the optimal MIC for the PROMIS-UE v1.2 [10]. Q30: Only the study of Randall et al. described the MIC for the PROMIS-UE. Two methods were used (MDCM and 0.5 SD method), which resulted in comparable MICs of 4.6 and 4.8. Q45: Median of 4.6 and 4.8 = 4.7.

Only one study reported the MIC for the PROMIS-UE v1.2, with MIC values of 4.6 and 4.8 (on a T-score metric, with a mean of 50 and SD of 10, higher scores indicate better function) (Table 2D) [24]. The study scored 4 out of 5 core credibility items. The optimal MIC value was 4.7 (Table 3 and Fig. 5). No studies were found on the MIC of the PROMIS-UE v2.0 or v2.1 in UEI patients, and scores from v1.2 cannot be compared to scores from v2.0 and v2.1.

## Discussion

This study illustrated a step-by-step selection approach for selecting an optimal MIC value for five commonly used PROMs for patients with UEIs [10]. No studies were found estimating the MIC for the MHQ and for the PROMIS-UE v2.0/v2.1 in UEI patients. Based on studies with high credibility, recommended MIC values are 9.4 for the DASH, 7.4 for the Q-DASH, 11.5 for the PRWE and 4.7 for the PROMIS-UE v1.2.

A variety of MIC values was found for each PROM. It is difficult to find explanations because the patient samples, sample sizes, time interval, anchor questions, and MIC methods used varied, and were often chosen not optimally. For the DASH, for example, the mean age of the study populations varied. However, the number of studies and patients was too small to study the influence of age on the MIC. Also, for the DASH the smallest study (n = 20) reported the highest MIC of 13.0, compared to the optimal value of 9.4, suggesting the validity of this MIC could be questioned [22].

Interestingly, lower MIC values (3.5–11.7) were found for the Q-DASH compared to the DASH (6.7 to 13.0), while the Q-DASH is a derivative of the DASH and scores are on the same metric [31]. While all three studies on the DASH included patients with humeral fractures (shoulder region), the studies on the Q-DASH included patients with elbow injuries. A possible explanation for the lower MICs in patients with elbow injuries is that these patients experience major limitations and therefore consider a small improvement already important.

For the PRWE, MIC values varied widely (9.2–56.0) and seemed very high. However, there is no explanation why the MIC of the PRWE would be higher than the MIC of the other PROMs. The optimal MIC value, based on two studies with the highest credibility was 11.5, indicating that the high MIC values may be biased.

To obtain valid and reliable MIC estimates, the patient sample and study design should be chosen carefully. Ideally, the study population should contain patients who changed as well as patients who did not change during the follow-up period. The subgroup who changed should ideally include a substantial number of patients who slightly changed, to reliably determine the threshold between improved and not improved patients. We recommend that these two subgroups both include at least 50 patients, based on COSMIN recommendations for responsiveness studies that have a similar design [32]. Finally, the time interval between baseline and follow-up should not be too long because patients may have difficulty recalling their previous health state. This requires knowledge of the study population and interventions and careful planning of the assessment moments.

Of major importance for the credibility of MIC estimates is the validity of the anchor question. The anchor and the PROM should measure the same underlying construct. For example, when the PROM measures upper extremity function, the anchor should ask about a change in upper extremity function. The recall period of the anchor question should match the time interval between baseline and follow-up. The validity of the anchor is reflected in the correlation between the PROM change score and the anchor. This correlation should at least be 0.30 but preferably above 0.50 to obtain a credible MIC estimate. This correlation is, however, often not reported, making it unclear if published MIC values are valid.

Another important factor determining the credibility of the MIC estimate is the MIC estimation method used. Three studies used distribution-based methods [27–29]. However, distribution-based methods refer to measurement error (minimal detectable change), rather than an important change for patients [4]. Furthermore, most of the anchor-based methods used are currently considered sub-optimal. Most MIC estimates were based on the mean change in PROM scores in patients who indicate ‘slightly improved’ on the anchor question. These MIC estimates are often based on a small (sub)sample. In addition, these MIC values represent the average of the group that is slightly improved, rather than the lowest value of this group. This means that the MIC value is probably overestimated [1]. This can be seen in the study of Ibounig et al., who reported a mean change MIC of 11.2, while the predictive modelling MIC was 9.4 [20]. The ROC method provides more reliable MIC estimates but the ROC MIC value is biased when the percentage of patients who importantly changed is not 50% [7]. This was observed in MIC studies of the PRWE. In many studies the percentage of improved patients was not reported, so the published MIC values may be biased. More sophisticated methods, such as predictive modelling, longitudinal IRT, or longitudinal CFA methods are currently recommended to determine the MIC [6–9].

The time interval between the baseline and follow-up measurements is also important to consider. For example, the highest MIC values (43.1 and 56.0) were found in studies with measurements at baseline (day 0) and 6 or 12 weeks after UEI [26]. However, the percentage of improved UEI patients between day 0 and week 6 or 12 will be much higher than 50%, since basically all trauma patients improve after the day of injury, indicating that these high MIC values are likely overestimations. A more appropriate time interval would be between weeks 6 and 12, which might explain why studies comparing weeks 6 and 12 found more credible MIC values of 14.6 and 11.5 [28, 30].

### Strengths and limitations

A strength of this study was a critical appraisal of the credibility of published MIC values [18]. In addition, we used a systematic step-by-step decision tree to select an optimal MIC value for each PROM, taking the credibility into account, but also the consistency of MIC values and the possible influence of contextualized factors [10].

A limitation of our study was that the credibility tool does not take the limitations of the mean change method and ROC method into account. Furthermore, there is overlap between the core criterion “Has the anchor shown good correlation with the PROM?” and the additional criteria about the correlation between the anchor and the PROM scores at baseline, follow-up, and change, respectively, which means that studies with low correlations between the anchor and the PROM may have been “punished” twice. Other limitations were the relatively few studies that could be included, and the heterogenous MIC methods and patient populations used in the included studies, limiting the generalizability of the proposed MIC estimates. Furthermore, the interventions applied could have been a contextual factor explaining the variability of MIC values. However, there were not enough most credible estimates to analyze contextualized factors. Finally, we did not include people with lived experience (UEI) in the design and analyses of the study because of the methodological nature of the study.

### Future perspectives

This study showed that published MIC values of the included PROMs vary widely, and MIC studies suffer from methodological flaws and lack of reporting, making it difficult to explain differences and select the wheat from the shaft. We believe that this applies to many other PROMs and MIC studies as well. The method of Wang et al. helps to select optimal MIC values among those published. However, there is room for improvement of this selection method. And more important, there is room for improvement in the quality and reporting of MIC studies.

Regarding the PROMs included we found no studies that determined a MIC for the MHQ in patients with UEI. This is remarkable because the MHQ is widely used in patients with hand and wrist injuries and even recommended by the ICHOM Hand and Wrist Conditions Standard Set [33]. We recommend future studies on the MIC of the MHQ in patients with UEI. Furthermore, the PROMIS-UE v2.0/v2.1 item bank, short forms and CAT are increasingly used PROMs for patients with UEI [16, 34, 35]. We found only one study estimating the MIC for the PROMIS-UE v1.2 in patients with UEI [24]. However, due to different metrics, this MIC value is not valid for versions v2.0 and v2.1. Future studies should be conducted to determine the MIC of the PROMIS-UE v2.0/v2.1 in patients with UEI. More high quality studies on the MIC of all included PROMs are recommended in patients suffering UEI, using valid anchors, a sufficient sample size, and an appropriate MIC method.

### Recommendations for research and clinical practice

While the method of Wang et al. presents a useful way to select an optimal MIC value, there is room for further improvement of this method, especially regarding the MIC credibility assessment. The credibility tool can be improved by taking the limitations of the mean change method and ROC method into account and remove the overlap between two criteria. The latter point was addressed by Wang et al. in a later paper, in which they proposed an extension of the credibility tool by adding an item addressing construct proximity as an alternative to the correlation item [36]. The criterion on MIC precision could be further strengthened by including a sample size recommendation per subgroup, rather than for the total study. Furthermore, the criterion on the optimal time interval could be strengthened by addressing not only the length of follow-up but also the intervention. One of the included MIC studies on the PRWE showed that a longer time interval is sometimes better than a short time interval [26]. Finally, it would be useful to develop a scoring algorithm to quantify risk of bias in MIC studies, for application in systematic reviews.

MIC values of PROMs can facilitate better interpretation of the effects of treatment and rehabilitation for patients with UEI in clinical practice. The optimal MIC values reported in this study can function as a guideline for healthcare providers in daily practice. Healthcare providers should be aware of a possibly relevant change when the change score of the patient is as high or exceeds the recommended MIC values of 9.4 for the DASH, 7.4 for the Q-DASH, 11.5 for the PRWE and 4.7 for the PROMIS-UE v1.2. However, one should keep in mind that every patient has their own unique MIC value and the relevance of change scores should always be checked with the individual patient [1]. In research, the MIC values can be used to determine the number of ‘responders’ (i.e. patients with a change score larger than the MIC) on group level [1]. However, MIC values should be interpreted with caution, because most MIC methods used do not account for measurement error of the anchor and the PROM. Only more recently developed methods, such as longitudinal IRT, take measurement error into account [8].

## Conclusion

This study illustrated a step-by-step approach to select the optimal MIC value for a PROM, taking the credibility of the available MIC studies, the correlation of the PROM change score with the anchor, the consistency of MIC values, and contextual factors (e.g. type of intervention) into account. The optimal MIC values selected are 9.4 for the DASH, 7.4 for the Q-DASH, 11.5 for the PRWE and 4.7 for the PROMIS-UE v1.2. More high quality studies on the MIC of these PROMs are recommended, using valid anchors, a sufficient sample size, and an appropriate MIC method. This study serves as an example of how the optimal MIC value can be carefully selected for a given PROM if multiple MIC values are reported in the literature.

## Supplementary Information

Below is the link to the electronic supplementary material.

Supplementary file 1 (DOCX 50 KB)

## Data Availability

All data generated or analysed during this study is included in this published article and its tables, figures and appendices.
