# Supplementary material for: Selecting the optimal MIC for five patient-reported outcome measures in patients with upper extremity injuries, using a systematic search and step-by-step decision tree
Source: Qual Life Res. 2026 Mar 13;35(4):103. doi: 10.1007/s11136-025-04152-1 (PMC12987879; doi:10.1007/s11136-025-04152-1)
Supplement: Supplementary file 1 — (DOCX 50 KB) [file 11136_2025_4152_MOESM1_ESM.docx]

**Appendix 1**: Search strategy in PubMed^1^

| **PROMs** | "Disability arm shoulder"[tiab] OR DASH[tiab] OR patient rated wrist evaluation*[tiab] OR PRWE[tiab] OR patient rated wrist hand evaluation*[tiab] OR PRWHE[tiab] OR Michigan hand questionnaire*[tiab] OR MHQ[tiab] OR Michigan hand outcomes questionnaire*[tiab] OR Michigan hand outcome questionnaire*[tiab] OR MHOQ[tiab] OR "Patient reported outcomes measurement information system"[tiab] OR "PROMIS"[tiab] |
| --- | --- |
| **MIC** | ((minimum[tiab] OR minimal[tiab] OR minimally[tiab] OR minimaly[tiab] OR clinical[tiab] OR clinically[tiab]) AND (important[tiab] OR significant[tiab] OR detectable[tiab] OR resolvable[tiab]) AND (change*[tiab] OR difference*[tiab])) OR MIC value[tiab] OR MICs value[tiab] OR MCID[tiab] OR MCIDs[tiab] OR (small*[tiab] AND (real[tiab] OR detectable[tiab]) AND (change[tiab] OR difference[tiab])) |
| **NOT** | ("address"[Publication Type] OR "biography"[Publication Type] OR "comment"[Publication Type] OR "directory"[Publication Type] OR "editorial"[Publication Type] OR "festschrift"[Publication Type] OR "interview"[Publication Type] OR "lecture"[Publication Type] OR "legal case"[Publication Type] OR "legislation"[Publication Type] OR "letter"[Publication Type] OR "news"[Publication Type] OR "newspaper article"[Publication Type] OR "patient education handout"[Publication Type] OR "popular work"[Publication Type] OR "congress"[Publication Type] OR "consensus development conference"[Publication Type] OR "consensus development conference, nih"[Publication Type] OR "practice guideline"[Publication Type]) NOT ("animals"[MeSH Terms] NOT "humans"[MeSH Terms]) |

*^1^An updated selection of relevant search terms from the validated search filter for finding studies on measurement properties was used.(1)*

*Reproduction of the search strategy can be achieved through combining the different sets (PROMs and MIC) with the Boolean operator AND. The search terms in Embase and the Cochrane Library were derived from the search terms used in PubMed and are available on request from the author.*

**Appendix 2:** Terms in literature for the MIC:

Minimum Important Change, Minimal Important Change, Minimally Important Change, Clinical Important Change, Clinically Important Change, Minimum Significant Change, Minimal Significant Change, Minimally Significant Change, Clinical Significant Change, Clinically Significant Change, Minimum Detectable Change, Minimal Detectable Change, Minimally Detectable Change, Clinical Detectable Change, Clinically Detectable Change, Minimum Resolvable Change, Minimal Resolvable Change, Minimally Resolvable Change, Clinical Resolvable Change, Clinically Resolvable Change, Minimum Important Difference, Minimal Important Difference, Minimally Important Difference, Clinical Important Difference, Clinically Important Difference, Minimum Significant Difference, Minimal Significant Difference, Minimally Significant Difference, Clinical Significant Difference, Clinically Significant Difference, Minimum Detectable Difference, Minimal Detectable Difference, Minimally Detectable Difference, Clinical Detectable Difference, Clinically Detectable Difference, Minimum Resolvable Difference, Minimal Resolvable Difference, Minimally Resolvable Difference, Clinical Resolvable Difference, Clinically Resolvable Difference, MIC value, MICs value, MCID, MCIDs, Smallest Real Change, Smallest Real Difference, Smallest Detectable Change, Smallest Detectable Difference.

**Appendix 3:** PROM characteristics

| DASH | 30 items (addressed to measuring disabilities and symptoms in UEI). Timeframe: during the last week.  Six different 5-point Likert response metrics:   - No difficulty/Mild difficulty/Moderate difficulty/Severe difficulty/Unable - Not at all/Slightly/Moderately/Quite a bit/Extremely - Not limited at all/Slightly limited/Moderately limited/Very limited/Unable - None/Mild/Moderate/Severe/Extreme - No difficulty/Mild difficulty/Moderate difficulty/Severe difficulty/So much difficulty that I can’t sleep - Strongly disagree/Disagree/Neither agree or disagree/Agree/Strongly agree.   Higher scores imply more disability: 0 (no disability) to 100 (most severe disability).(2) |
| --- | --- |
| Q-DASH | 11 items. The shortened version of the DASH. All items are also included in the full DASH.  The Q-DASH follows the same response metrics, total scores and timeframe.(3) |
| PRWE | 15 items (addressed to pain and function after wrist injury). Timeframe: during the last week.  Two different 11-point response metrics:   - Pain: 0 (no pain) to 10 (unbearable pain) - Function: 0 (no disability) to 10 (most disability)   Higher scores imply worse outcome: 0 (no disability and pain) to 100 (most disability and unbearable pain).(4) |
| MHQ | 57 items (addressed to 6 subgroups; activities of daily living (ADL), overall hand function, pain, work performance, aesthetics and patient satisfaction with their hand function). Timeframe: during the last week.  Six 5-point Likert response metric:   - Very good/Good/Somewhat good/Bad/Very bad - Not difficult at all/A little difficult/Somewhat difficult/Moderately difficult/Very difficult. - Always/Often/Sometimes/Rarely/Never - Very mild/Mild/Moderate/Severe/Very severe - Strongly agree/Agree/Neither agree nor disagree/Disagree/Strongly disagree - Very satisfied/Somewhat satisfied/Neither satisfied nor dissatisfied/Somewhat dissatisfied/Very dissatisfied   The total score per metric is converted to a score ranging from 0 to 100. Higher scores imply higher disability, pain and dissatisfaction: 0 (normal situation) to 100 (worst situation).(5) |
| PROMIS-UE v2.0 item bank | 46 items (addressed to UE function). Timeframe: ‘current status’.  Two 5-point Likert response metrics:   - Unable to do/With much difficulty/With somewhat difficulty/With a little difficulty/Without any difficulty - Cannot do/Quite a lot/Somewhat/Very little/Not at all   Scores are expressed as T-scores, with a mean of 50 and SD of 10 in the general population.  Higher scores indicate better function.(6, 7) |
| PROMIS-UE v2.0 SF | 7 items (addressed to UE function). These 7 items are part of the full item bank.  One 5-point Likert response metric:   - Unable to do/With much difficulty/With somewhat difficulty/With a little difficulty/Without any difficulty   The PROMIS-UE v2.0 SF uses the same scoring and timeframe as the PROMIS-UE v2.0 item bank.(6, 7) |
| PROMIS-UE v2.0 CAT | 4-12 items (addressed to UE function).  CAT requires 4 to 12 items to complete, with an average of 5 items.(7)  Two 5-point Likert response metrics:   - Unable to do/With much difficulty/With somewhat difficulty/With a little difficulty/Without any difficulty - Cannot do/Quite a lot/Somewhat/Very little/Not at all   The PROMIS-UE v2.0 CAT uses the same scoring and timeframe as the PROMIS-UE v2.0 item bank.(6, 7) |

**References**

1. Terwee CB, Jansma EP, Riphagen, II, de Vet HC. Development of a methodological PubMed search filter for finding studies on measurement properties of measurement instruments. Qual Life Res. 2009;18(8):1115-23.

2. Hudak PL, Amadio PC, Bombardier C. Development of an upper extremity outcome measure: the DASH (disabilities of the arm, shoulder and hand) [corrected]. The Upper Extremity Collaborative Group (UECG). Am J Ind Med. 1996;29(6):602-8.

3. Franchignoni F, Vercelli S, Giordano A, Sartorio F, Bravini E, Ferriero G. Minimal clinically important difference of the disabilities of the arm, shoulder and hand outcome measure (DASH) and its shortened version (QuickDASH). J Orthop Sports Phys Ther. 2014;44(1):30-9.

4. Walenkamp MM, de Muinck Keizer RJ, Goslings JC, Vos LM, Rosenwasser MP, Schep NW. The Minimum Clinically Important Difference of the Patient-rated Wrist Evaluation Score for Patients With Distal Radius Fractures. Clin Orthop Relat Res. 2015;473(10):3235-41.

5. Chung KC, Pillsbury MS, Walters MR, Hayward RA. Reliability and validity testing of the Michigan Hand Outcomes Questionnaire. J Hand Surg Am. 1998;23(4):575-87.

6. Terwee CB, Roorda LD, de Vet HCW, Dekker J, Westhovens R, van Leeuwen J, et al. Dutch-Flemish translation of 17 item banks from the Patient-Reported Outcomes Measurement Information System (PROMIS). Quality of Life Research. 2014;23(6):1733-41.

7. van Bruggen SGJ, Lameijer CM, Terwee CB. Structural validity and construct validity of the Dutch-Flemish PROMIS((R)) physical function-upper extremity version 2.0 item bank in Dutch patients with upper extremity injuries. Disabil Rehabil. 2019:1-9.

**Appendix 4: MIC credibility scores**

| **Author, year** | **Item 1 *core***  **(high/low)** | **Item 2 *core***  **(high/low)** | **Item 3 *core***  **(high/low)** | **Item 4 *core***  **(high/low)** | **Item 5 *core***  **(high/low)** | **Total core (high/total)** | **Item 6 *add***  **(high/low)** | **Item 7 *add***  **(high/low)** | **Item 8 *add***  **(high/low)** | **Item 9 *add***  **(high/low)** | **Total add**  **(high/total)** | **Total score**  **(high/total)** |
| --- | --- | --- | --- | --- | --- | --- | --- | --- | --- | --- | --- | --- |
| **Huyke-Hernández et al. 2023 [7]** | high | high | low | high | high | 4/5 | low | low | low | low | 0/4 | 4/9 |
| **Ibounig et al.**  **2022 [1]** | high | low | high | high | high | 4/5 | low | low | low | low | 0/4 | 4/9 |
| **Iordens et al. 2017 [4]** | high | high | low | low | high | 3/5 | low | low | low | low | 0/4 | 3/9 |
| **Larose et al.**  **2024 [8]** | low | low | low | high | low | 1/5 | low | low | low | low | 0/4 | 1/9 |
| **Mahabier et al. 2017 [2]** | high | high | low | low | high | 3/5 | low | low | low | low | 0/4 | 3/9 |
| **McCreary et al. 2020 [9]** | high | low | low | low | low | 1/5 | low | low | low | low | 0/4 | 1/9 |
| **Perez-Ubeda et al. 2024 [10]** | low | low | low | high | low | 1/5 | low | low | low | low | 0/4 | 1/9 |
| **Randall et al. 2021 [5]** | high | high | low | high | high | 4/5 | low | low | low | low | 0/4 | 4/9 |
| **Walenkamp et al. 2015 [11]** | high | high | low | low | high | 3/5 | low | low | low | low | 0/4 | 3/9 |
| **Wanstrom et al. 2024 [6]** | high | high | high | high | high | 5/5 | high | low | low | low | 1/4 | 6/9 |
| **van de Water et al. 2014 [3]** | high | high | low | low | high | 3/5 | high | low | low | low | 1/4 | 4/9 |

**References**

1. Ibounig, T., et al., *Minimal important difference and patient acceptable symptom state for common outcome instruments in patients with a closed humeral shaft fracture - analysis of the FISH randomised clinical trial data.* BMC Med Res Methodol, 2022. **22**(1): p. 291.

2. Mahabier, K.C., et al., *Reliability, validity, responsiveness, and minimal important change of the Disabilities of the Arm, Shoulder and Hand and Constant-Murley scores in patients with a humeral shaft fracture.* J Shoulder Elbow Surg, 2017. **26**(1): p. e1-e12.

3. van de Water, A.T., et al., *Reliability and validity of shoulder function outcome measures in people with a proximal humeral fracture.* Disabil Rehabil, 2014. **36**(13): p. 1072-9.

4. Iordens, G.I.T., et al., *Minimal important change and other measurement properties of the Oxford Elbow Score and the Quick Disabilities of the Arm, Shoulder, and Hand in patients with a simple elbow dislocation; validation study alongside the multicenter FuncSiE trial.* PLoS One, 2017. **12**(9): p. e0182557.

5. Randall, D.J., et al., *The minimal clinically important difference of the Patient-Reported Outcomes Measurement Information System (PROMIS) physical function and upper extremity computer adaptive tests and QuickDASH in the setting of elbow trauma.* JSES Int, 2021. **5**(6): p. 1132-1138.

6. Wanstrom, J., et al., *The minimal important difference and smallest detectable change of the Oxford elbow score, Quick disabilities of the arm shoulder and hand and single assessment numeric evaluation used for elbow trauma.* JSES Int, 2024. **8**(4): p. 897-902.

7. Huyke-Hernandez, F.A., et al., *The Minimum Clinically Important Difference for the Patient-Rated Wrist Evaluation in Surgical Fixation of Distal Radius Fractures: Does Hand Dominance Make a Difference?* Injury, 2023. **54**(10): p. 110959.

8. Larose, G., et al., *Trajectory of Recovery following ORIF for Distal Radius Fractures.* J Wrist Surg, 2024. **13**(3): p. 230-235.

9. McCreary, D.L., et al., *Interpreting Patient-Reported Outcome Results: Is One Minimum Clinically Important Difference Really Enough?* Hand (N Y), 2020. **15**(3): p. 360-364.

10. Perez-Ubeda, M.J., et al., *Adjuvant Arthroscopy Does Not Improve the Functional Outcome of Volar Locking Plate for Distal Radius Fractures: A Randomized Clinical Trial.* Arthroscopy, 2024. **40**(2): p. 305-317.

11. Walenkamp, M.M., et al., *The Minimum Clinically Important Difference of the Patient-rated Wrist Evaluation Score for Patients With Distal Radius Fractures.* Clin Orthop Relat Res, 2015. **473**(10): p. 3235-41.
